# ProLint: a web-based framework for the automated data analysis and visualization of lipid–protein interactions

**DOI:** 10.1093/nar/gkab409

**Published:** 2021-05-26

**Authors:** Besian I Sejdiu, D Peter Tieleman

**Affiliations:** Centre for Molecular Simulation and Department of Biological Sciences, University of Calgary, 2500 University Drive NW, Alberta T2N 1N4, Canada; Centre for Molecular Simulation and Department of Biological Sciences, University of Calgary, 2500 University Drive NW, Alberta T2N 1N4, Canada

## Abstract

The functional activity of membrane proteins is carried out in a complex lipid environment. Increasingly, it is becoming clear that lipids are an important player in regulating or generally modulating their activity. A routinely used method to gain insight into this interplay between lipids and proteins are Molecular Dynamics (MD) simulations, since they allow us to study interactions at atomic or near-atomic detail as a function of time. A major bottleneck, however, is analyzing and visualizing lipid–protein interactions, which, in practice, is a time-demanding task. Here, we present ProLint (www.prolint.ca), a webserver that completely automates analysis of MD generated files and visualization of lipid–protein interactions. Analysis is modular allowing users to select their preferred method, and visualization is entirely interactive through custom built applications that enable a detailed qualitative and quantitative exploration of lipid–protein interactions. ProLint also includes a database of published MD results that have been processed through the ProLint workflow and can be visualized by anyone regardless of their level of experience with MD. The automated analysis, feature-rich visualization, database integration, and open-source distribution with an easy to install process, will allow ProLint to become a routine workflow in lipid–protein interaction studies.

## Graphical Abstract

**Graphical Abstract:**
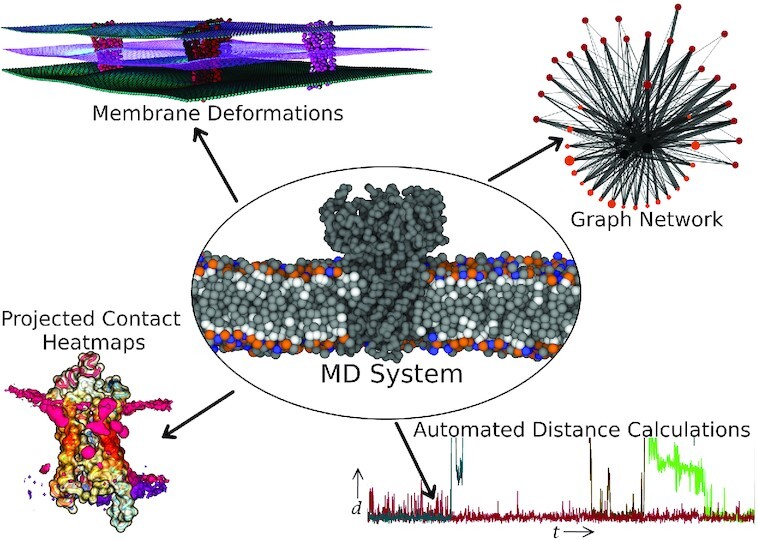
We present ProLint (www.prolint.ca), a webserver that completely automates analysis of MD generated files and visualization of lipid–protein interactions.

## INTRODUCTION

Effective communication of scientific results is contingent on the careful analysis of the collected data and its display in a condensed but still information rich format. This is particularly the case in computational studies, such as Molecular Dynamics (MD) simulations, where even using conservative output-control parameters tera-bytes of data are continuously generated (1,2). When studying interactions between membrane proteins with their surrounding lipid environment, MD simulations have proven to be an excellent tool to probe many details of their interplay that are inaccessible to experiments. The process of analyzing the resulting data, however, becomes an arduous task especially when the datasets are large, or the story they tell complex. Similarly, presentation of results is restricted within the tight confines of journal rules in terms of type and number of graphical elements and best judgement on the part of the authors on what aspect of their work is most interesting. This inefficient data analysis process coupled with a fragmented visualization leads to a wasteful use of time and computer resources as well as unintentional biases when publishing results (3,4).

Parallel to the increase in the computing power of modern hardware and development of more efficient software, MD simulations continue to increase in both size and time scale. Concurrently, there has been an ever-growing number of freely available software that facilitates manipulation of MD output formats (coordinates, trajectories, and topology files). MDTraj (5) and MDAnalysis (6) are two frequently used packages that provide specialized tools for trajectory manipulation and various analysis methods, including distance calculation and neighbor searching. Simulation packages such as GROMACS (7), AMBER (8), CHARMM (9) and NAMD (10,11) also come pre-packaged with a set of tools for users to manipulate and analyze output files, as well as dealing with many package-specific tasks. These are, however, general purpose tools requiring significant manual customization for specific analysis workflows, which is usually done through custom, usually un-shareable, scripts.

**Figure 1.**
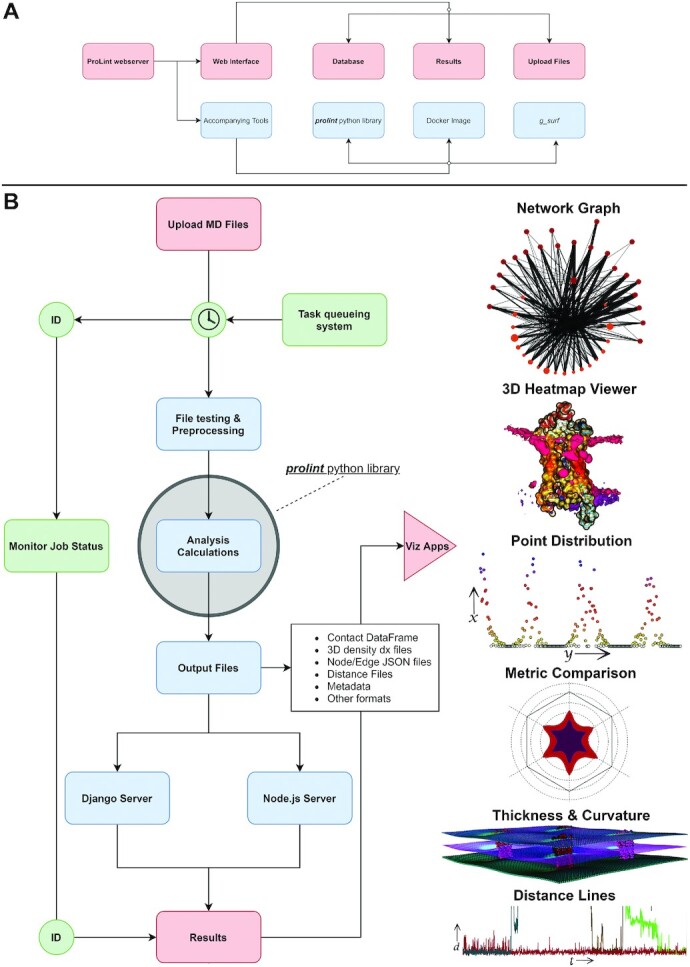
The ProLint webserver. (**A**) The webserver along with the different tools released alongside it: a multi-image Docker build that includes the entire source code of ProLint and can be easily installed locally, a stand-alone python library that also includes the visualization applications, and g_surf. (**B**) The workflow implemented by ProLint: submissions are put into a task queueing system and once the required computer resources become available, they are preprocessed and analyzed through an automated protocol. Results are stored for 24 hours and can only be accessed through a unique ID provided to the user upon submission. The server is hosted on an AWS EC2 instance with an Elastic IP address and secure HTTPS protocol enabled using Certbot. Database management is done using PostgreSQL hosted on AWS RDS and files are stored using AWS S3. Task queueing is done using the Celery software. The backend is build using Django and Node.js, whereas the frontend uses HTML, CSS and JavaScript. Docker is used to provide an easy to install instruction set to run everything in a closed-system locally or host it on a private network.

Here, we introduce ProLint (www.prolint.ca), an open-source webserver that aims to bridge the widening gap between data generation and gaining insight into biologically relevant lipid–protein interactions. It automates the process of analyzing MD output files for lipid–protein interactions and provides a dedicated fully featured application platform to visualize results. All applications are interactive, providing the user with many convenient ways and options to explore the underlying dataset regardless of the size and complexity. The workflow implemented by ProLint, depicted in Figure 1, and summarized further below, completely automates the process of analyzing and visualizing lipid–protein interactions from MD generated files. ProLint is open-source and can also be easily installed and run locally.

## MATERIALS AND METHODS

### Automated analysis and visualization of lipid–protein interactions

Detailed reviews of lipid–protein interactions show that analysis methods can be conceptually grouped into three categories: contact analysis, density distribution, and physics-based methods (12,13). Contact-based analysis relies on defining a particular spatial distance between lipids and protein residues as them being in *contact* and then using different metrics to quantify those contacts. Density calculations highlight lipid–protein interactions by quantifying the preferential localization of lipids in either the membrane plane or in three dimensions. Physics-based methods measure the changes experienced by the lipid environment caused by the presence of membrane proteins. This usually includes measurements of membrane thickness and curvature profiles. ProLint analysis includes methods from all categories. Importantly, it aims to be as comprehensive as possible in its analysis workflow and to rely on visualization applications to offload tasks such as data selecting and filtering which can be done interactively by the user (Figure 1 and Supplementary Figure S1). ProLint has been tested using data that employ the Martini model (14,15), widely used to study membrane proteins and lipid systems, but presently also includes the ability to upload data from atomistic simulations in a beta-feature and will support atomistic simulation very soon.

### A modern UI/UX interface with an included database

ProLint implements a modern, secure and responsive frontend that runs on most web browsers. The visualization framework leverages modern and highly popular interactive visualization libraries: bokeh, Three.js, D3.js (16) and NGL Viewer (17,18) to visualize server-side calculated analysis results. The backend uses a task queuing system to keep track of submitted jobs until required computer resources become available. Each submission is associated with a unique ID that can be used to access results. ProLint also implements its own database where we host precalculated results of data that has undergone a prior peer review process. We hope that this curated and growing dataset is going to serve the wider scientific community with an interest in lipid–protein interactions, including researchers who may lack either the technical knowledge of MD simulations or coding experience. The modern frontend, intuitive visualization platform and included database significantly extend the reach and usability of ProLint. Lastly, the frontend also includes detailed documentation, highlighting the analysis and visualization applications, along with tutorials and several example datasets.

### The ProLint workflow to lipid–protein interactions

ProLint is an entirely web-based approach to lipid–protein interactions. However, we also provide stand-alone versions that can be run locally. The entire source code for ProLint is made available through an open-source license (https://github.com/ProLint/ProLint) and the entire webserver can be run locally using the provided Docker application by simply downloading the repository and executing:

### Docker-compose up

This single command will automatically build the Linux containers and python environment required for ProLint to run on any machine. We also provide a stand-alone python package called *prolintpy* (https://github.com/ProLint/prolintpy) that can be used to further customize the workflow by enabling users to easily include, for instance, additional metrics (e.g. residence time), fine-tune analysis and automate the workflow. In terms of the analysis performed, there is some similarity between *prolintpy* to the PyLipID library (https://github.com/wlsong/PyLipID) (19). However, while they both calculate various contact-based metrics, PyLipID aims more at identifying and ranking binding sites with lipids, whereas *prolintpy* is less assuming about the actual binding sites and aims more at providing a comprehensive analysis (with support for density measurements, contact heatmaps and physics-based methods) and leave the choice of binding site identification to the user by using a rich interactive visualization platform. An important feature of *prolintpy* is that it supports all of the visualization features of ProLint, which it does by using the JupyterLab environment. *prolintpy* uses MDTraj (5) to read data files and compute neighboring atoms.

Finally, we also provide *g_surf (**20**)*, a powerful tool that we have used to calculate density, thickness, and curvature profiles (21). This is important because this type of analysis is an aspect of lipid–protein interactions that has been lacking in the literature as most studies focus on only the specific binding of a few lipids. g_surf is written in C, but we also provide a python wrapper script that can be used to interface with it. As a platform, ProLint aims at being agnostic with respect to the computational model used and as such it works with both atomistic and coarse-grained models.

### Requirements and restrictions

The ProLint webserver limits the file size of data that can be uploaded, and it reads the input data using a submission form. Users who require more detailed customization should use the prolintpy package whereas users who wish to analyze large systems are encouraged to install ProLint locally. Before submission, data files must contain only protein and lipids (no water or ions atoms/beads). For density calculation, proteins must be centered before they are uploaded. Protein centering can be done using any of the trajectory processing tools that come by default with the major simulation packages.

## RESULTS

Below, we describe three representative examples of the capabilities of ProLint.

### Specific binding of cholesterol to the Smoothened receptor

**Figure 2.**
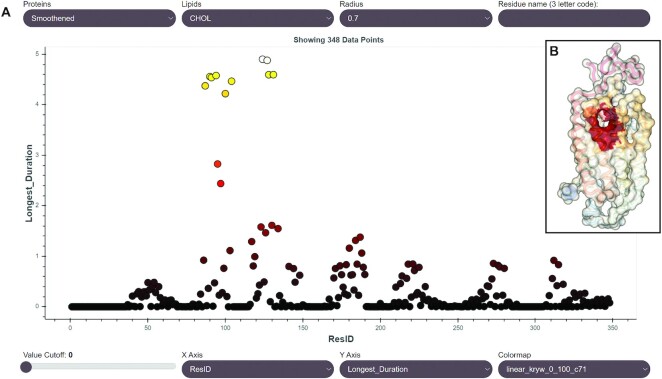
Cholesterol interactions with Smoothened. (**A**) Cholesterol interacts specifically with Smoothened by tightly binding between the helices 2 and 3 of the receptor. This is clearly observable from the distribution of contact points. Hovering over the data points reveals its labeling information. (**B**) Contact heatmap of the same data points but projected on the surface of the receptor, thus accurately localizing the binding site. Supplementary Figure S2 provides a more detailed overview of the contact heatmaps supported by ProLint.

Cholesterol binding to Smoothened has been noted in several structural and biochemical studies. Cholesterol is integral to the functioning of Smoothened and has been observed to completely enter the 7-helical core of the receptor (22,23). The mechanistic details of this modulation are unknown. MD simulations, at different levels of resolution, have been used to probe the interaction profile of Smoothened with membrane lipids (24). An example trajectory of such a simulation is available for testing here: (https://www.prolint.ca/resources/data). With it as input, ProLint generates a detailed interaction profile that, among others, includes the visualization shown in Figure 2. Smoothened displays one clearly defined binding site for cholesterol that is located between helices 2 and 3 of the receptor. In fact, this matches exactly with what has been reported from previous MD studies of the same receptor (24–26) including the accurate identification of the same residues noted there. ProLint allows you to visualize contacts with lipids by projecting them onto the surface of the protein and to switch between different metrics as well as visualize lipid densities at different contour levels (Supplementary Figures S2 and S3).

### The distinct binding modes of cholesterol and PIP lipids

**Figure 3.**
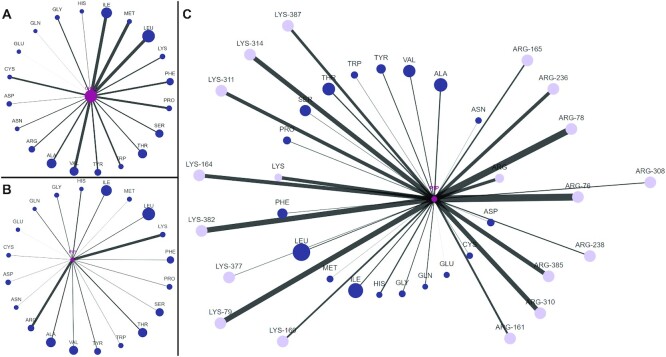
Cholesterol and PIP lipids interact through different mechanisms. (**A**, **B**) The interaction network of cholesterol and PIP lipids with the serotonin (5HT1B) receptor, respectively. (**C**) An expanded view of serotonin-PIP lipid interaction profile showing all arginine (ARG) and lysine (LYS) residues. The size of lipid nodes represents their total fraction of all lipids in the system, whereas residue node sizes are based on their relative fraction that make up the serotonin receptor. Edge width visualizes the average number of contacts with each lipid type. It is easy to focus over each node and to switch between different metrics.

Figure 3 shows how interactive network graphs can be used to display and compare the different interaction levels of lipids with residues based on their physicochemical properties. We can easily show, for example, the well-known fact that cholesterol and PIP lipids, while both interacting preferentially with proteins, do so by preferring to associate with quite different residues (12,27): cholesterol interacts with hydrophobic residues (Figure 3A) with the additional involvement of aromatic residues (e.g. phenylalanine); PIP lipids, on the other hand, interact mainly with positively charged residues (Figure 3B). Since the application is interactive, it is possible to expand each residue node to reveal the interaction of each lipid type with every single residue making up the node (Figure 3C). This shows that we can gain deeper insight into the data that extends far beyond simple interaction dynamics.

### GPCR family-wide visualization of lipid–protein interactions

**Figure 4.**
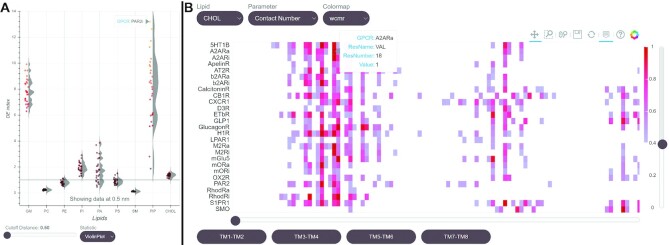
Integrating datasets and gaining novel insight. (**A**) The enrichment of different lipids around GPCRs. Showing how some lipids are highly enriched while other are depleted. (**B**) Sequence heatmap of GPCR–lipid interactions across all the GPCR data showing a likely conserved binding site at the extracellular site of helices 6 and 7.

ProLint provides a freely explorable database of lipid–protein interactions. This example demonstrates how this can be useful to gain new biological insight from available data. Figure 4 shows two example applications for 28 different GPCRs taken from ref. (25). Figure 4A shows the diversity of lipid enrichment around GPCRs and the striking differences in how, for example, PIP lipids interact with them. Figure 4B shows an interactive sequence heatmap of aligned GPCR structures. When we look at interactions with cholesterol, for instance, we can see that almost all class A GPCRs (but not non-class A GPCRs) display a conserved binding site at the extracellular interface between helices 6 and 7. These results clearly show the deep biological insight enabled by freely and responsively visualizing big datasets spanning many proteins. These applications can be build using data from the ProLint database and the code that can be used for their visualization is made available on the ProLint repository (https://github.com/ProLint). All of the GPCR data is freely available on the webserver along with a commitment to support other groups who wish to make their data publicly available as well.

## DISCUSSION

The iterative process of data analysis and visualization is often time consuming. It is not uncommon in studies of lipid–protein interactions, for analysis and visualization to take more time than production runs of MD simulations, which is already a lengthy process. ProLint aims at bridging this gap between data generation and gaining valuable insight about a system through the following four core objectives:

1. **Automated and modular analysis:** Analysis is always context-dependent, and users will invariably have varying levels of interest for different aspects of the same data. ProLint recognizes this and both the analysis on the backend and visualization on the frontend implement a modular approach to lipid–protein interactions.
2. **Interactive visualization**: It is impossible to automate visualization without making many strict assumptions on what data to display and in what format. ProLint overcomes this limitation by heavily focusing on building application with which users can interact and explore their data however they choose.
3. **Automation & scalability**: Analysis and visualization of lipid–protein interactions is entirely automated. ProLint also provides a standalone python library that users can use to automate tasks and scale-up their workflow.
4. **Accessibility & shareability**: Web-based visualization and cloud-based hosting allow ProLint to share MD results with the whole scientific community. Thanks to its intuitive visualization applications that require no coding knowledge or MD experience, the benefit of the shared data extends far beyond the computational community.

The line between data analysis and data visualization becomes indistinguishable once users can interact with the data being presented. The design and development of the ProLint webserver is centered around automated and comprehensive analysis of lipid–protein interactions and user interactivity. We think these features will enable ProLint to become a routine workflow when working with lipid–protein interactions.

## DATA AVAILABILITY

ProLint is open-source and available through the following GitHub page: https://github.com/ProLint.

## Supplementary Material

gkab409_Supplemental_File

## Notes

Present address: Besian I. Sejdiu, Department of Structural Biology and Center for Data Driven Discovery, St Jude Children's Research Hospital, Memphis, TN, USA.
