# Supplementary material for: ProLint: a web-based framework for the automated data analysis and visualization of lipid–protein interactions
Source: Nucleic Acids Res. 2021 May 26;49(W1):W544–50. doi: 10.1093/nar/gkab409 (PMC8262751; doi:10.1093/nar/gkab409)
Supplement: gkab409_Supplemental_File [file gkab409_supplemental_file.docx]

ProLint: a web-based framework for the automated data analysis and visualization of lipid-protein interactions

Besian I. Sejdiu^1*^, D. Peter Tieleman^1*^

^1^ Centre for Molecular Simulation and Department of Biological Sciences, University of Calgary, 2500 University Drive NW, Alberta, T2N 1N4, Canada

Present Address: Besian I. Sejdiu, Department of Structural Biology and Center for Data Driven Discovery, St Jude Children’s Research Hospital, Memphis, TN, USA

SUPPLEMENTARY INFORMATION


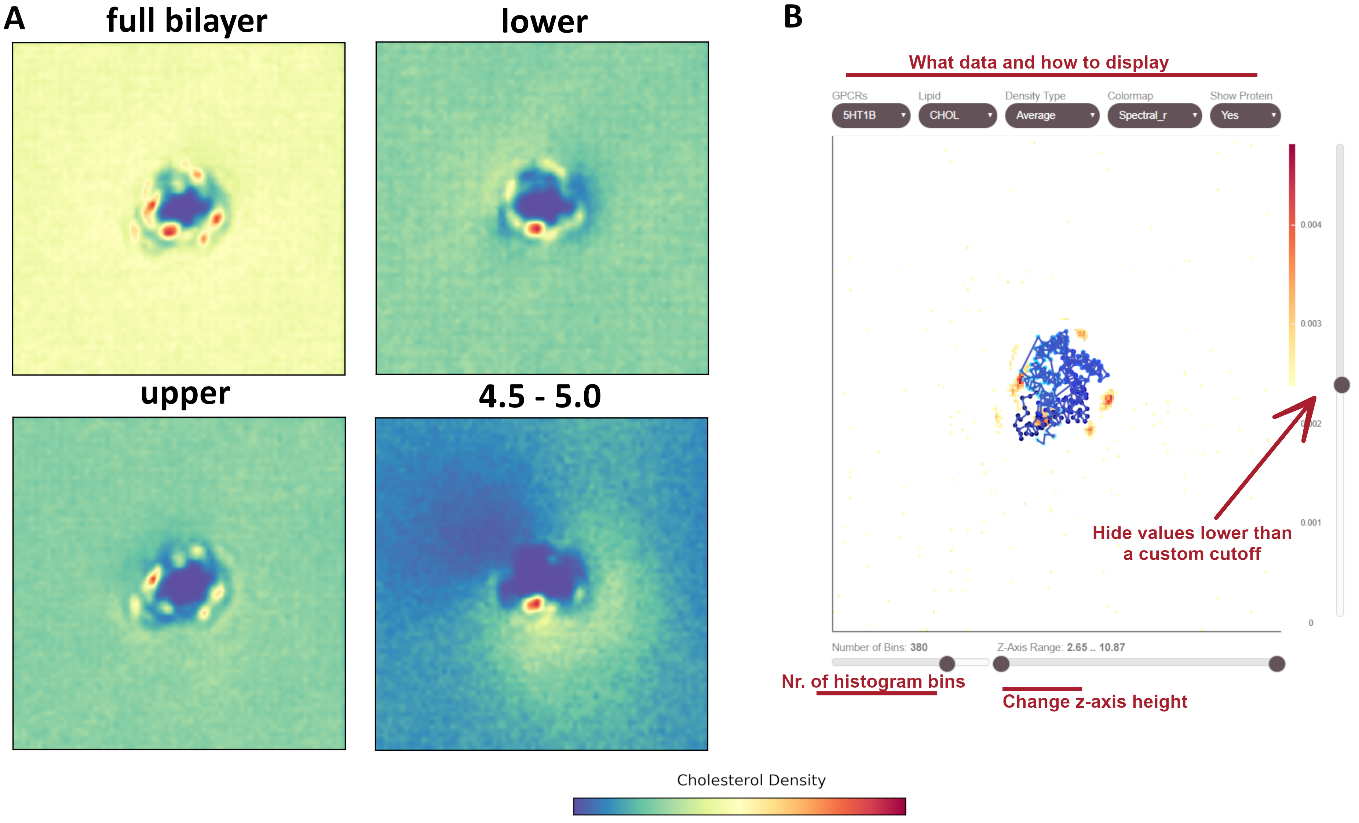


**Figure S1. 2D density profile analyses of cholesterol-protein interactions for the Serotonin Receptor (5HT1B).** **A.** Conventional 2D density profiles calculated for the full-length bilayer, for the lower and upper membrane leaflets as well as for a small subsection of the lower layer (4.5 – 5.0 nm). **B.** ProLint application showing the 2D density profile with different options available to change the way the density is calculated and visualized with real time analysis and feedback. The right-most slider allows for a subset of the data to be hidden to easily distinguish localization sites according to their density, regardless of the colormap employed.

**An example use-case:** The density profile of the full bilayer gives a rough idea of the localization of cholesterol molecules in the system. A common approach used in the literature is to separate the analysis for the upper and lower leaflet, but this is still insufficient to accurately pinpoint the position of these potential binding sites. Figure 4B show a ProLint application that fixes many of these shortcomings. The application allows for the visualization of all proteins in the system as well as options to change the number of bins and coloring scheme used in the calculation of the density profile. There is also the option to visualize the protein which makes it a lot easier to estimate the location of the interaction sites as well as a slider to change the normal (z) axis range of the bilayer to the desired width. In this example, a user could easily pinpoint the location of the interaction site with cholesterol in Figure 4 to between 4.5 – 5 nm of bilayer height.


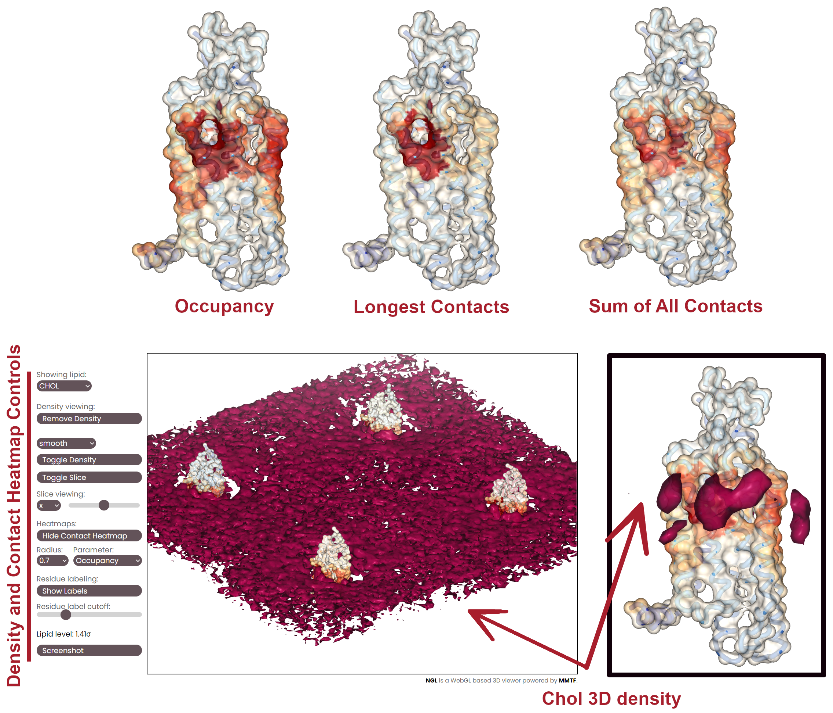


**Figure S2. Interactive application to visualize contact heatmaps and densities.** The top row shows a coloring of protein residues according to their interaction level with lipids as measured by three different commonly used metrics: the sum of all contacts of each residue with cholesterol, the longest contacts, and occupancy. The bottom row gives a full view of the ProLint-provided application along with the different options and controls.

**An example use-case:** Identifying and defining binding sites with lipids is complicated. Results are heavily dependent on what metric we use to measure interactions, and as such it is crucial that we have a broad and comprehensive view of the lipid-protein interactions happening in our system. Using ProLint you can switch between the different metric profiles by a simple selection through a dropdown menu. You can visualize the spatial density of the lipid of interest (cholesterol in the figure, but you can easily change the lipid too). You can then fine-tune the density by changing the isolevels, which gives you the image shown on the right with highly localized cholesterol distributions.


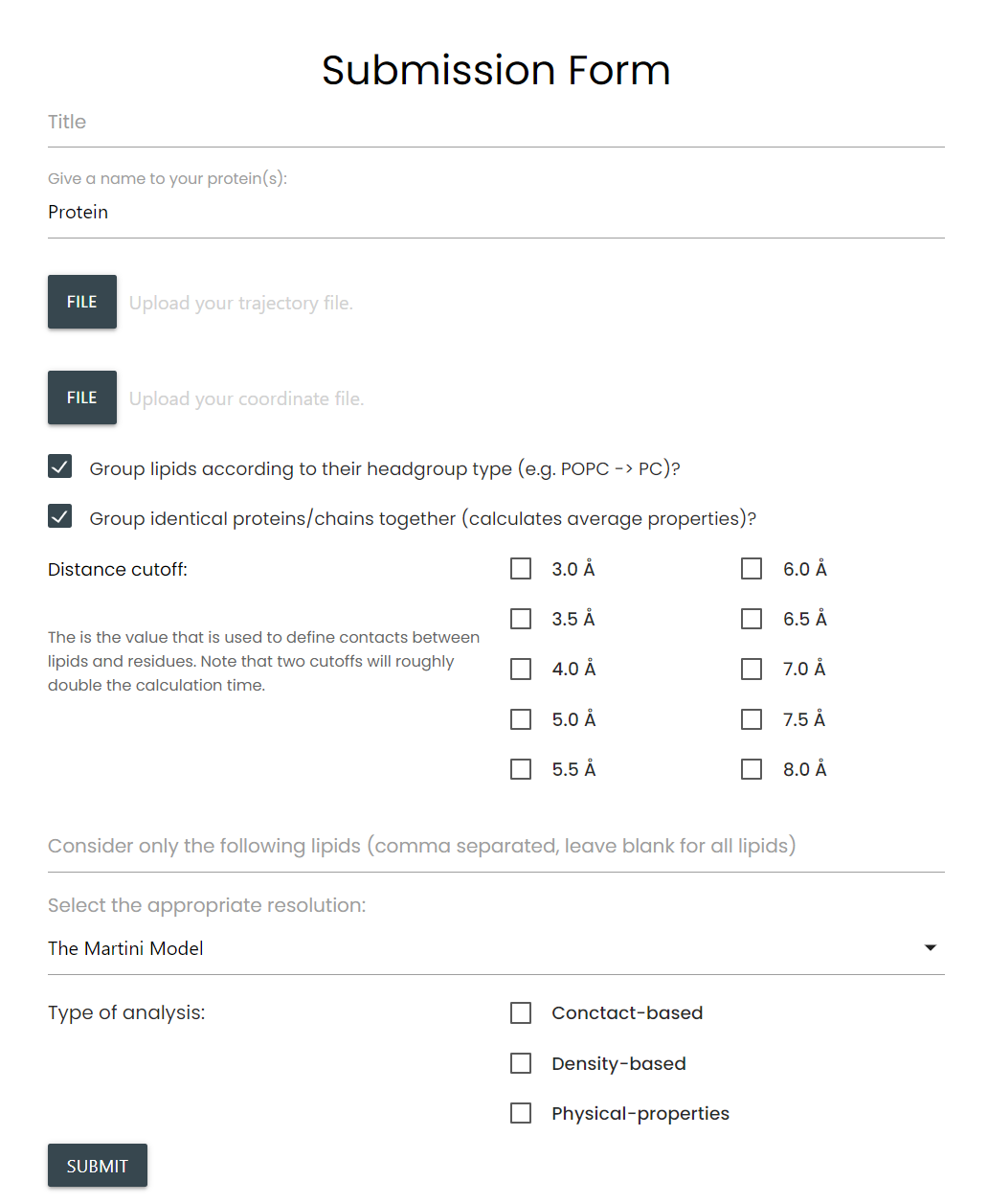


**Figure S3. The Submission Form used by ProLint.** The form is well-documented and supports input validation to ensure correct submission. Users can also choose the resolution of data they submit and specify the type of analysis to perform. This modular approach to analysis makes it easy to extend the capabilities of ProLint, by for instance supporting the recently released Martini 3 model, add new and different analysis protocols, etc.

**Software Use**

ProLint relies on and builds on several open-source libraries. In addition to the visualization libraries pointed out in the main text, the ProLint framework also uses, among others, the following libraries: numpy(1), scipy(2), pandas(3), matplotlib(4), colorcet(5), networkx(6), django, Jupyter Notebooks(7), nglview (8), gridDataFormats (part of MDAnalysis)(9), celery, etc.

**References**

1. Harris, C.R., Millman, K.J., van der Walt, S.J., Gommers, R., Virtanen, P., Cournapeau, D., Wieser, E., Taylor, J., Berg, S. and Smith, N.J. (2020) Array programming with NumPy. *Nature*, **585**, 357-362.

2. Virtanen, P., Gommers, R., Oliphant, T.E., Haberland, M., Reddy, T., Cournapeau, D., Burovski, E., Peterson, P., Weckesser, W. and Bright, J. (2020) SciPy 1.0: fundamental algorithms for scientific computing in Python. *Nat. Methods*, **17**, 261-272.

3. McKinney, W. (2011) pandas: a foundational Python library for data analysis and statistics. *Python for High Performance and Scientific Computing*, **14**, 1-9.

4. Hunter, J.D. (2007) Matplotlib: A 2D graphics environment. *Comput. Sci. Eng.*, **9**, 90.

5. Kovesi, P.D. (2000) MATLAB and Octave functions for computer vision and image processing. *Centre for Exploration Targeting, School of Earth and Environment, The University of Western Australia, available from:* [*http://www*](http://www)*.csse.uwa.edu.au/∼pk/research/matlabfns*, **147**, 230.

6. Hagberg, A., Swart, P. and S Chult, D. (2008). Los Alamos National Lab.(LANL), Los Alamos, NM (United States).

7. Kluyver, T., Ragan-Kelley, B., Pérez, F., Granger, B.E., Bussonnier, M., Frederic, J., Kelley, K., Hamrick, J.B., Grout, J. and Corlay, S. (2016) *Jupyter Notebooks-a publishing format for reproducible computational workflows*.

8. Nguyen, H., Case, D.A. and Rose, A.S. (2018) NGLview–interactive molecular graphics for Jupyter notebooks. *Bioinformatics*, **34**, 1241-1242.

9. Gowers, R.J., Linke, M., Barnoud, J., Reddy, T.J.E., Melo, M.N., Seyler, S.L., Domanski, J., Dotson, D.L., Buchoux, S. and Kenney, I.M. (2019). Los Alamos National Lab.(LANL), Los Alamos, NM (United States).
